# Bringing the home into school: learning and connecting through mathematics education during the time of a pandemic

**DOI:** 10.1007/s10649-022-10157-1

**Published:** 2022-05-25

**Authors:** Jodie Hunter, Roberta Hunter, John Tupouniua, Generosa Leach

**Affiliations:** Institute of Education, Massey University, Auckland, New Zealand

**Keywords:** Home-school partnerships, Culturally sustaining mathematics pedagogy, Equity, Primary education, Teacher learning

## Abstract

The COVID-19 pandemic brought with it a new way of being in a changed and uncertain world. Aotearoa/New Zealand took a well-being approach and in turn, we share the positive outcomes which resulted for some low socio-economic schools and communities in relation to teacher learning and relationships with families. In this article, we report on how teachers and schools connected with diverse students and their families during the period of remote learning. We draw on the responses from 20 teachers and school leaders who participated in interviews. Following the wider government focus, schools took a well-being first approach which led to increased connections and positive home/school relationships. The results highlight how a disruptive event such as COVID-19 can also be a time to focus on strengths of diverse communities and gain insights. We demonstrate that while focusing on mathematics, teachers and school leaders gained insights related to their students’ funds of knowledge and saw opportunities for learning for students, parents, and the teachers themselves.

## Introduction

The COVID-19 pandemic is something nobody in Aotearoa/New Zealand had ever experienced or expected to experience in their lifetime. As a closely connected and interrelated diverse population of people, the choice was taken by our political leaders and scientists that as a small island nation we needed to adapt our daily lives to focus on collective well-being (Bourke et al., 2021). This widely accepted position was one which focused on positioning lives as more important than wealth (Giovannetti, 2020). Within this frame, as researchers and educationalists we were provided with another positive opportunity; specifically, the chance to reinvent how mathematics education might look outside the traditional constraints of the formal structures of schools and classrooms. Being able to reimagine and learn how mathematics teaching and learning can happen differently is particularly pertinent for Māori and Pasifika, who have been traditionally underserved in mathematics classrooms in Aotearoa/New Zealand. Māori are indigenous to New Zealand, while Pasifika people are closely related to Māori. They are a diverse and heterogeneous group of people and include recent arrivals from many island nations (e.g., Samoa, Tonga, Cook Islands, Niue, Tokelau, Fiji, Solomon Islands, Tuvalu) as well as multiple generations born in New Zealand (Anae et al., 2001).

For too long, Māori and Pasifika learners within schools in Aotearoa/New Zealand have experienced structural inequities. These have resulted in lowered levels of mathematical achievement (May et al., 2019). The reasons are many and with layers of complexity, but one key reason can be attributed to the increasingly diversified populations of students in classrooms in the twenty-first century which in Aotearoa/New Zealand has compounded the long reaching negative effects of colonization. This has emphasized the need for teachers to engage with students whose home lives and context differ from their own and which Rubie-Davies (2016) signals has potential to cause cross-cultural misunderstandings. In our prior work, we have drawn on culturally sustaining (Paris, 2012) and strength-based approaches (González et al., 2005; Hunter & Hunter, 2018) to support teachers to successfully engage in these poly-ethnic mathematics classrooms. We have done this by explicitly deepening their knowledge of the lived realities of their students’ home contexts (Hunter et al., 2019). But this new novel COVID-19 pandemic environment has opened the doors further and allowed us to position homes squarely within school communities. In doing so, we have all become learners, researchers, school leaders, and teachers alike, as we have grappled with working with students and their families with mathematics in a remote online situation. In this paper, we want to share the learnings we have all made. Within the specific setting of homes becoming part of school during a period of lockdown, the questions we aim to explore are (1) How do teachers develop and maintain culturally sustaining mathematics pedagogy during remote teaching and learning? (2) What did teachers learn as they remotely “entered” students’ homes during the pandemic?

### Culturally sustaining mathematics pedagogy

Culturally sustaining mathematics pedagogy (CSMP) is a form of teaching mathematics long supported by multiculturalists (e.g., Banks et al., 2009; Nieto & Bode, 2011) because it espouses an education system that is both culturally diverse and equitable. Such an education system has, at its core, a curriculum which is culturally sustaining, with corresponding appropriate pedagogical and assessment practices organized so that interactions are facilitated across racial and ethnic lines (Johnson, 2014). The way in which we view culturally sustaining pedagogy builds on the seminal work of Gay (2002, 2010) and Ladson-Billings (1994, 1995). In the following subsections below, we highlight some of the hallmarks of CSMP that guide and inform our study.

#### A strength-based approach to mathematics education

Building on the frame of culturally responsive and sustaining pedagogy allows us to position our work as a strength-based approach for mathematics education. Within this perspective, it is argued that students learn more successfully when their prior knowledge and cultural contexts serve as the basis for their learning (Adiredja, 2019; Bannister et al., 2018; Jilk, 2016; Rogoff et al., 2017). These researchers argue that sophisticated skills and resources for learning mathematics among marginalized populations have been identified, but these skills have often been misinterpreted through deficit perspectives. For example, Ruvalcaba et al. (2016) unveiled how a nuanced and sophisticated form of fluid collaboration among Mexican–American students helped them engage with an advanced task in computer programming. The authors claimed that while this nuanced form of collaboration was novel from a dominant cultural perspective, it was a cultural norm among the Mexican students. Hence, for Māori and Pasifika students, effective learning of mathematical practices and discourses views their cultural practices and values as assets rather than liabilities (Hunter et al., 2016; Si’ilata, et al., 2018). This strength-based perspective supports us to sustain “linguistic, literate and cultural pluralism as part of the democratic project of schooling and as a needed response to democratic and social change” (Paris & Alim, 2014, p. 88). Inherent within this perspective are beliefs related to empowerment of the whole community involved within our mathematics classrooms. In the next section, we discuss the importance of connecting to and affirming the funds of knowledge of students and their families.

#### Funds of knowledge—connecting learning with home and community experiences

In alignment with a strength-based approach, the theoretical framing of funds of knowledge affirms that all peoples and cultures have “historically accumulated and culturally developed bodies of knowledge and skills essential for household or individual functioning and well-being” (Moll et al., 1992, p. 133). Teachers who are aware of student, family, and community funds of knowledge can then use these to mathematize students’ lived experiences in meaningful ways. More generally, a funds of knowledge perspective of mathematics strives to connect the curriculum and pay respect to students’ lived experiences at home, school, and their wider communities.

Bonner (2014), while investigating the practices of successful teachers of underserved students in the USA, found that parents and community members valued teachers who supported students to take pride in their cultural identity and retain cultural practices while learning mathematics. For example, one teacher successfully engaged students in her class by drawing on student experiences and practices within a Black church that many attended and connected to this in the mathematics classroom with the use of music and movement. Bonner highlighted that teacher awareness of student and family funds of knowledge was an important component in relationship building.

#### Drawing on students’ cultural values and experiences to establish a safe and respectful learning environment

As alluded to above, incorporating students’ cultural values in the teaching and learning of mathematics is fundamental to CSMP. Past research studies show that improved equitable educational outcomes in mathematics can be achieved for Māori and Pasifika students when their cultural values are acknowledged and respected in the classroom (Anthony, 2013; Civil & Hunter, 2015; Hunter, 2021). Classrooms that sustain students’ cultural values are ones in which students feel safe to be who they are, to take mathematical risks in their reasoning and are grounded in their cultural identities.

Pasifika people, while a heterogenous group, share a set of common values (Ministry of Education, 2013). According to the Pasifika Education Plan developed by the New Zealand Ministry of Education, the core Pasifika values are belonging, family, inclusion, leadership, love, reciprocity, relationships, respect, service, and spirituality. These Pasifika values parallel core Māori values such as whanaungatanga (sense of belonging), kotahitanga (oneness), tuakana/teina (relationships), kaitiakitanga (reciprocity), whakapapa (family lineage), aroha (love), wairua (spiritual well-being), and hauora (see Berryman & Eley, 2017; White, 2011). Evident in these values is the strong role of collectivism along with service and well-being. Many research studies (e.g., Anthony, 2013; Hill et al., 2019; Hunter, 2021) demonstrate that these collectivist-orientated values remain important to students while learning mathematics. For example, two different studies (e.g., Hill et al., 2019; Hunter et al., 2019) found that Pasifika students ranked family, respect, and collectivism as key mathematics educational values important to their learning. Others (e.g., Civil & Hunter, 2021; Hunter & Civil, 2021) show how these values play out in classrooms which actively affirm them. In the final section of this review, we explore the importance of home-school relationships and how these impact not only the ways in which students engage with mathematics but also their cultural identity.

#### Developing respectful relationships between school, families, and the wider community

Another key aspect of CSMP is the development of respectful, reciprocal relationships between educators, students, and their families and communities. Research literature both from within New Zealand and internationally (e.g., Averill, 2012; Bonner, 2014; Hunter et al., 2016; Robinson et al., 2009) highlights both the importance of teaching that aligns with cultural values and experiences along with parental involvement in schooling. Within notions of empowerment, the development of shared-power relationships and trust are of paramount importance (e.g., Bonner, 2014; Civil & Hunter, 2015; Hodge & Cobb, 2016; Powell et al., 2016). In the present study, we consider empowerment and power sharing as not only being related to the students and the wider collective of community members but also to teachers and school leaders. One of our aims will be to explore the way in which the building of relationships and trust across the whole collective occurred and how power sharing developed as students, their families, and their teachers engaged in mathematics.

Studies (e.g., Barton et al., 2004; Civil & Bernier, 2009; Monson, 2010) also show that parent involvement and engagement can have significant effect on students’ mathematics achievement and dispositions. However, Barton et al. (2004) argue that traditional notions of “parental involvement” need to be expanded because they are often measured from a school perspective. This means that parents (especially from minority low-income families) whose involvement does not resemble traditional forms of involvement are classified in the literature as having minimal involvement. Civil and Bernier (2009) suggested that a wider view of effective partnerships between schools and families considers language, individual differences, and parental concerns, and views parents as partners in the education process.

Within the Aotearoa/New Zealand context during the COVID-19 pandemic lockdown, a study by Riwai-Couch et al. (2020) surveyed the views of parents of Māori and Pasifika primary and secondary students. The key findings were that while parents enjoyed the freedom to decide on learning content as well as how and when learning took place, many were concerned with how children would re-adapt to the structures and expectations of school when they returned. This reflected their ongoing unease related to home-school partnerships. In another report undertaken during the initial lockdown, Bourke et al. (2021) highlight how many of the children they interviewed from minoritized cultures made links to how their extended length at home with extended family provided them with opportunities to immerse themselves in their own culture. What this report showed was the importance they placed on being in a culturally congruent space. These researchers note that the lockdown enabled these children not only to recognize how their culture differed from the dominant culture of Aotearoa/New Zealand, but also to “reconnect with and strengthen learning through language, culture and identity” (p. 30). The challenge inherent in both these reports is how mathematics educators can learn from these findings and develop better home-school partnerships for all students.

In the next section, we describe the research context that this research is grounded in.

### Research context: developing mathematical inquiry communities

Developing mathematical inquiry communities (DMIC) was founded within an evidence-based professional learning and development (PLD) model. Of importance in this PLD is the emphasis placed on gaining equitable outcomes for all students (Civil & Hunter, 2015; Civil et al., 2019; Hunter et al., 2018). Set in Aotearoa/New Zealand, DMIC PLD utilizes culturally sustaining pedagogy within ambitious teaching (Kazemi, et al., 2009) and complex instruction (Featherstone et al., 2011). Within this model, systematically marginalized students (e.g., Māori, Pasifika, and other diverse students) are positioned to have opportunities to learn mathematics with deep reasoning using a range of mathematical practices (Hunter & Anthony, 2011). Heterogeneous grouping across levels of achievement rather than streamed ability groups are used as culturally appropriate for our Māori and Pasifika learners (Hunter & Hunter, 2018; Hunter et al., 2020a, b). Other research (e.g., Alton-Lee et al., 2011; Hunter, 2021; Hunter et al., 2019, 2020a, b) also shows that student wellbeing, cultural identity, mathematical identity, motivation, and self-esteem have been enhanced by engaging in learning mathematics in classrooms where teachers are participating in the DMIC PLD. DMIC PLD focuses on building collaborative learning communities across and within schools and classrooms, and with groups of teachers and teacher educators.

## Methodology

To understand the context of the initial lockdown within Aotearoa/New Zealand, we begin with a brief timeline of the pandemic. Our first case of COVID-19 was reported on February 28th, 2020. By March 21st 2020 with a rising number of cases, a four-level alert system was introduced by the Aotearoa/New Zealand government to help combat COVID-19. On March 23rd 2020 with over 100 cases and evidence of community transmission, Aotearoa/New Zealand moved to a higher alert level 3 and educational facilities were closed. At midnight on March 25th, 2020, the country moved into the highest alert level 4 and all non-essential businesses closed with people instructed to stay at home for a minimum of a 4-week lockdown period. Aotearoa/New Zealand schools were closed from March 25th, 2020 until a partial reopening when the country moved to alert level 3 on April 28th, 2020. Approximately 5–10% of students returned to school. The full reopening of schools after the initial lockdown occurred on the 18th of May, 2020 after the country moved to alert level 2. The data included in this study is from a larger study examining the experiences of mathematics teacher educators, mathematics teachers, and school leadership during the initial COVID-19 pandemic lockdown period in Aotearoa/New Zealand.

In this article, we focus on the responses of 20 primary school educators as they reflect on their experiences during the COVID-19 pandemic lockdown in Aotearoa/New Zealand. An invitation was sent by the first author to 30 schools who were engaged in the DMIC PLD and were using online teaching methods. In total, 15 teachers and five principals/deputy principals from 15 different schools agreed to participate. Most of the participants (*n* = 18) taught at urban schools in low socio-economic areas with high numbers of Pasifika and Māori students. The other two participants taught at schools in a rural location with a range of students from different socio-economic backgrounds but including a significant percentage of Māori students. The cultural background of the participants was predominantly Pakeha (European) (*n* = 15), with three participants who were Pasifika, one who was Māori, and one who was Indian. All of the participants, aside from one, had worked at their respective school for 2 years or longer and all participants were qualified teachers with between 3 and 28 years teaching experience.

### Data collection methods

In this study, we were interested in exploring educators’ experiences, perceptions, and learning as they connected with students and their families during the remote learning period in Aotearoa/New Zealand. Data collection used semi-structured interviews of between 30 and 45 min that were conducted through Zoom. The rationale for using interviews as the sole data collection method was three-fold. Firstly, data collection for this study took place when the country was in lockdown conditions. Audio-recorded interviews conducted through Zoom were a means of connecting safely with participants. Secondly, interviews provide opportunities to gather detailed information in relation to the participants’ beliefs, perspectives, and experiences (Flick, 2018; Seldman, 2019). This aspect is directly connected to the research aim of this article to learn from educators’ perspectives. Thirdly, the semi-structured interview process allowed us to make sense of the participants’ points of views, and check and verify our understanding of their perspectives.

For the overall study, 14 questions were used to collect data. These were related to educators’ overall experiences in teaching during the lockdown period; support provided from the Ministry of Education and the DMIC team; experiences in teaching mathematics and connecting with students and families; using digital platforms; and maintaining pedagogy aligned with DMIC PLD. Specifically, in this article, we focus on the responses gathered in relation to the following five interview questions:

1. How has this [teaching during a lockdown] gone for you?
2. What have been the barriers in reaching the parents and students in your school? How have you overcome any barriers? What has worked well for you?
3. What are the things that you need to consider when using Zoom or a digital platform to teach mathematics with your students at home?
4. What are the things that you have learnt about your students through using Zoom or another digital platform to teach mathematics?
5. What do you know about how parents have kept the learning for children in mathematics going? In what ways is this different than school?

### Data analysis

This study sought to explore and understand educators’ experiences, perceptions, and learning as they connected with students and their families during the remote learning period in Aotearoa/New Zealand. Thematic analysis was used to examine the data that was collected. Thematic analysis is a way of identifying themes across a data-set to make sense of collective or shared experiences (Boyatzis, 1998; Braun & Clarke, 2012). We used both an inductive and deductive approach to coding, with the codes and sub-codes developed from the interview data and previous research literature and policy documents. For example, sub-codes such as using multiple methods to connect with families were developed using an inductive approach, whereas the sub-codes related to using Pasifika values in pedagogy or promoting Pasifika language use were developed in a deductive way from earlier research studies.

In the first instance, the research team transcribed the interview responses, read these in full, and individually annotated the transcripts to identify initial codes and themes. Common codes and themes that appeared across responses to different questions were then identified and grouped together to develop patterns. Within Table 1, a definition of the umbrella code and examples of sub-codes are provided.

**Table 1:** Analytical framework and explanation

Umbrella code	Definition of umbrella code	Sub-codes
Building relationships with whanau (family)	Actions and activities which educators used to build or develop existing relationships with students and whanau	- Using multiple methods to connect (email, phone, social media) - Connecting to Pasifika values (e.g., showing respect in interactions, understanding the role of family) - Drawing on whanau and community connections - Consideration of family circumstances
Culturally sustaining mathematics pedagogy and remote teaching	Actions and activities related to remote teaching and CSMP	- Maintaining inclusiveness and safety for students - Choosing accessible strands of mathematics - Building on home contexts and activities - Explicitly using Pasifika values in pedagogy (e.g., collectivism, supporting and encouraging family members to work together) - Promoting and valuing use of home language
Teacher learning related to CSMP	Learning related to students, whanau, and CSMP from the period of remote teaching and learning	- Developing knowledge of students individually as people outside of the classroom - Recognizing individual student strengths (language, mathematical capability, disposition) - Recognizing whanau as assets

Data were coded independently by two members of the research team and then crosschecked. Throughout the process, the research team engaged in discussions of the coding and for instances where there were contradictions, a discussion was undertaken between the research team so that a consensus was reached.

Given the context of this study and focus on culturally sustaining pedagogy for Pasifika students and their families, it is important to note the experience and background of the research team. This team of four comprised two researchers who were of Cook Island heritage and one researcher of Tongan heritage, as well as one Pakeha researcher. This supported analysis in relation to Pasifika values and funds of knowledge. Additionally, three of the research team members have a teaching background within New Zealand schools. Insights gained from the interviews are presented in the following section, by drawing on multiple voices of the educators to frame what they learnt in bringing the home into school during the COVID-19 lockdown.

### Potential limitations of the study

One potential limitation on the generalizability of our study is the relatively small number of participants we interviewed (*N* = 20). Furthermore, our sample was self-selected in regard to including only those who accepted the invitation to participate and composed of particular groups—18 participants were from urban (as opposed to rural) schools, and 15 participants were Pakeha. Finally, a potential limitation stems from the fact that our data consisted solely of the views of educators. This aligned with our specific aim of exploring what educators had learned from remotely entering students’ homes. However, gaining a broader view of what we can learn from when homes become part of the “school context” requires future research to also gather data from the views of students and parents/caregivers.

## Findings and discussion

In the findings and discussion, we elaborate on each theme from the interviews with teachers and school leadership. We interrogate these themes and stories to focus on broader lessons in relation to how teachers developed and maintained culturally sustaining pedagogy in mathematics during remote teaching and learning. We also use this to address the key learnings from educators’ perspectives when they “entered” students’ homes as part of schooling during the pandemic.

### From a well-being orientation to building relationships

In the stories that educators told of connecting with students and their families throughout the lockdown, it was evident that the well-being of the community was a clear focus for both school leadership and teachers. They all described how they recognized the need to take a holistic view of the well-being of both students and families, before considering mathematics teaching. For example, one principal, Melani stated: “pastoral care for the community has been a real worry before worrying about the curriculum or academics. A big focus on the well-being of the family rather than the individual child.” Forms of pastoral care that were noted by school leadership included schools providing food packages to families in the few days before the lockdown began. This was described by a senior leader, Emma, as a means of both building and shifting relationships into a well-being orientation: “I think that helped build a bridge because it wasn’t us necessarily just checking up on learning, it was ‘this was a support for me and my family.’” In this way, connections were made to core Pasifika values of respectful relationships and reciprocity. Within a Pasifika frame, reciprocity aligns with generosity and the act of sharing with or gifting to others to help them (Funaki, 2016).

Well-being, as a key aspect of education, stayed foremost and educators were cognizant that this was a stressful and anxious time for many families. For example, Stephanie described: “I had to step back and realize that everyone was going through times at the moment and I didn’t know what was going on for each family.” Every participant described reaching out to connect with parents and families in supportive ways. This included avoiding overloading families with messages. For example, being aware that Pasifika families often live in larger multi-generational households, many schools allocated family groups to a teacher rather than having multiple teachers contacting the same family*.* The educators drew on numerous modes of communication to ensure that they stayed connected with whanau. This included conventional means such as phone calls, text messages, and email. Other descriptions included the use of social media platforms such as Facebook and TikTok to connect with their school community. Also key, was building on existing connections between peer groups, different whanau and the wider community. This included asking people in the same vicinity of the neighborhood to check on families in a safe, socially distanced way and as Jenn stated: “reaching out to our Samoan, Tongan, Kiribati community, the leaders in that community to get them to contact people for us.” In this way, relationships were strengthened and provided a foundation for educators to begin learning with and from families.

In regard to mathematics teaching and learning, an approach was taken which drew on a philosophy of being empathetic to family circumstances and conscious of the differing priorities and challenges for families. To support this, many educators maintained connections in a supportive way with no expectation that students had to complete work or engage with online mathematics material. As Lily stated, this approach of connecting, supporting, and prioritizing well-being meant: “that most families have kept an open line of communication with us. No one feels bad if all they got done that week was one maths challenge or only just managed to get to the whole class zoom.” The actions of the teachers and schools to ensure well-being could best be described as encompassing the Māori concept of hauora*.* As they attended to the physical, mental and emotional, social, and spiritual well-being of their communities, they brought a strengthened relationship through bringing the home into the school. This goes some way towards what Barton et al. (2004) argue for in changing the school-home partnership.

By positioning student and whanau well-being at the center, we argue that the initial COVID-19 pandemic lockdown in Aotearoa/New Zealand provided an opportunity to develop stronger relationships between students and their whanau, teachers, and schools. As Riwai-Couch et al. (2020) argue, relationships are a key component needed to build a shared-power relationship between homes and schools, as is clearly illustrated in this study. All school participants recognized the valuable opportunities the pandemic provided them with to build these shared relationships. They stressed the importance of initial and ongoing contact as they were brought into their students’ homes through multiple means. This clearly showed that foundations for new ways of engaging across the school and home community were possible. Once the immediate well-being needs of the community were addressed, the teachers were then able to turn their attention to mathematical learning, again in a new space where they needed to go into their students’ homes through remote means.

### Taking mathematics into homes and opening spaces for mathematics through remote learning

Despite the challenges that the pandemic had brought to schools and communities, there was a sense that the teachers and school leaders all wanted to continue with the journey towards developing culturally sustaining mathematics pedagogy. It was evident that the teachers were mindful of how to enact responsive, inclusive pedagogy while teaching remotely. Many of the teachers reflected on whether synchronous teaching via Zoom resulted in more pressure for families who were doing shift work. Also of consideration was the need to maintain an inclusive space on Zoom and ensure positive student relationships. A number of teachers referred to the physical cues of relationships that were lost when trying to teach synchronously online. For example, Jo explained why she avoided using breakout rooms: “there are some dynamics in the classroom [pause] when you are sitting in the classroom, you can feel their body language, you just remove that with the breakouts.” This demonstrates the care that teachers were taking to maintain wairua (spiritual well-being), while also further developing relationships.

Acknowledging and drawing on students’ cultural values is a fundamental aspect of culturally sustaining mathematics pedagogy. Collectivism has a strong role in Pasifika culture, and the importance of family is a key value (MoE, 2013; Hunter, 2021). Both teachers and school leadership were able to consider how these values could be promoted when working in an online community with multiple members of whanau alongside students. A principal, Melani, recounted her teachers discussing whether collaborative heterogenous groups could be used: “I said, well we always talk about mixed ability groups, and when they have got older siblings around, in a way that is a mixed ability group.” Many teachers reflected positively on the way in which siblings, parents, and other household members joined in and interacted as a collaborative group during Zoom sessions. Clearly, for educators watching the productive cross-age group interactions extended their understandings of the richness of knowledge and capability that is brought to heterogenous groupings. Furthermore, they were able to view firsthand what Ruvalcaba et al. (2016) describe as nuanced sophisticated interactions enacted by their Māori and Pasifika students in their whanau setting. Through this, they were able to see possibilities embedded within the cultural values of their students which could be enacted in their own mathematics teaching. Other research has shown such interactions occurring within school or face-to-face situations, but in this study we have shown similar interactions within an online setting.

Similarly, being taken into student homes during the synchronous online mathematics lessons positioned teachers to reflect on their role as visitors into the students’ homes. For Pasifika people, home is vā, a sacred space. One teacher, Rajvir, noted the importance of: “acknowledging that you are a visitor into a family’s home, so it is a privilege to be there.” Another teacher, Tevita, took the mathematics classroom into the home saying:

We have that whanau component so that we are all together as a whanau. You realise I am manuhiri (visitor) in your house and you are manuhiri in my class. I am trying to show I value what they do. You don’t just go barreling in there saying ‘I’m the teacher do as I say’. It’s a role reversal being respectful as you are presenting yourself in their domain.

As student homes were brought into school settings, teachers reflected on forms of communication to maintain respectful relationships and acceptance that not all students and families would be comfortable with others’ viewing their homes. One school leader, Beth, specifically acknowledged that they decided to avoid synchronous platforms such as Zoom given that: “not all kids are in a position to have a quiet space where they can join a meeting.” Being able to accommodate students and whanau while considering cultural values provided the teachers with real learning about the lives of their students and whanau. Many researchers (e.g., Averill, 2012; Bonner, 2014; Powell et al., 2016) have shown the importance of such actions in building relationships of trust and respect.

Honoring and maintaining a student’s home language is an important aspect of culturally sustaining pedagogy. Language was a barrier for some teachers when taken into their students’ homes because of their monolingual background, in contrast to the students and families who were often multilingual and spoke both English and Pacific languages. However, others noted the richness they observed of children learning and practicing mathematics in their first language. They also recognized that not having tasks in other home languages precluded family involvement. As Liz, a teacher, stated:

The feedback we had from our school community is often the whole whanau is getting involved in the learning, and the inclusion of multiple languages would enable those families who don’t have strong English to be involved in learning as well.

These teachers were recognizing that to be truly culturally-sustaining, language is another aspect which needs to considered within an asset-based perspective (Paris & Alim, 2014) and that to develop a wider perspective of effective partnerships as promoted by Civil and Bernier (2009), multiple languages need to be honored.

To allow students to access mathematics in their homes, teachers carefully considered what would be engaging and accessible for their students. This included taking into account the strands of mathematics that would align with familiar activities and contexts for students and their whanau. In relation to position and orientation, one example drew on family and community engagement with gym training and sports, asking students to design an obstacle course for siblings or a fitness course for their family and to map this in a mathematical way. Commonly, teachers provided ideas to focus on practical measurement using area and perimeter. A school leader, Beth, described a video a whanau had posted of a year four student covering a table using books: “there is a really clever one [video excerpt] where she organises and measures with the book so there are no gaps and there is no overhang.” Notably, the teachers highlighted how parents contextualized these mathematical activities for their home life including contexts such as tiling a laundry room and baking food such as panipopo (coconut buns). The provision of these types of tasks and their design ensured that whanau members could work together on them through drawing on their collective funds of knowledge (Moll et al., 1992) and mathematising. These provided further reinforcement to educators of the value of culturally appropriate contextualized mathematics tasks.

Although the teachers provided students and their whanau with tasks, teachers also noted how parents were not always reliant on school-provided mathematics tasks. They described how families utilized other day-to-day activities to support mathematics learning. These included cooking, tiling, building and fixing things, and outdoor chores. A teacher, Jenn, recalled a parent describing her son’s activity:

He’s set up a firewood business with his cousins next door. They are collecting pine cones and he’s chopping kindling...it covers maths, working out the sales figures…and working out marketing/business plan.

Another teacher, Kate, who worked at a school in a rural area said: “One boy has been learning how to mix petrol and oils for a motorbike.” This was a part of the student’s home life in a rural farming area and the teacher recognized the potential for learning about ratios and proportions. It was clear that the teachers were being directly exposed to, and learning about, their student’s funds of knowledge (Moll et al., 1992) within mathematics. As Barton et al. (2004) explained, parental involvement can impact significantly both mathematical achievement and disposition. Being taken into the homes also changed the home-school partnership as teachers learnt the extent of parental knowledge of mathematics in their home settings. Civil and Bernier (2009) argue that this is needed if parents are to be viewed as true partners in the education process.

### Bringing the home into school: educators developing relationships and growing their understanding of culturally sustaining mathematics pedagogy through changing the boundaries

The remote teaching during the initial COVID-19 lockdown had challenges but also gave teachers an opening to notice their students’ strengths in relation to mathematics. Interestingly, the move away from the physical space of the classroom highlighted for some teachers the need to be conscious of how different levels of status had previously affected student participation during mathematics lessons. This included teachers such as Rita reflecting on how they noticed particular students “having to think and share their own voice,” rather than previous experiences in the classroom which had more uneven levels of participation with student status moderating who was speaking and which ideas were followed. Notably, as Justine acknowledged students that were described as shy and quiet: “have blossomed and shared their voice more.” For the teachers, their students had emerged as individuals within this changed setting, and this provided them with new perspectives of them.

Remote learning of mathematics both through synchronous and asynchronous means also provided opportunities for the educators to learn about student’s mathematical dispositions. A number of teachers commented on the resilience of their students in relation to learning at home. As Justine said: “I’ve learnt about how strong and intelligent they are to work through a difficult situation but still be able to access their learning.” Some teachers provided students with choices in relation to the difficulty of mathematical task levels, for example, Rita provided students with sets of decimals to regroup, compare, and order, and she noted that: “most students chose the more difficult ones.” This led to the reflection that the students were more capable than she thought: “they know way more maths than I thought they did.” In this way, teachers had their expectations of student capability challenged which set a platform for higher expectations when returning to the classroom, a key aspect of culturally sustaining mathematics pedagogy.

Importantly, the period of remote learning and bringing the home into the school context provided critically important opportunities to counter the deficit views many school educators construct in relation to Pasifika and Māori students and their whanau. These often occur through what Rubie-Davies (2016) describes as cross-cultural misunderstandings. Educators began to realize the importance that their school communities placed on mathematics. For example, one principal, Melani, while reflecting on the numbers of students and parents attending different lessons across the curriculum stated: “Maths would be one of the most popular zoom sessions. It shows parents place a lot of value on maths.” Observing families working together positioned teachers to reflect on their views of students and whanau. For example, Liz described how she had learnt: “How engaged some of our fanau (families) are in their children’s learning! I’m not sure if this is a new thing or has gone under the radar.” Another teacher, Jenn, explicitly identified previous bias and deficit theorizing:

it’s helped challenge our thinking, actually our families are really interested and invested in their children’s education. You know it’s just having some of those biases, you think you’re doing alright but you’re actually confronted around some of that. There is one family whose child I taught two years ago, and I found them really disengaged, I found them hard to get to places, and some of the excuses for why the child was away. I think now you can see them in their happy place and you can see the creativity and the risk taking, and the family involvement in the learning, so I guess for us it’s about learning about the children but also their wider family context.

The period of remote learning during the initial COVID-19 lockdown gave the educators opportunities to view whanau members’ interactions around learning mathematics. It appeared that this opportunity was a productive way to highlight strengths and challenge deficit perceptions. Hunter et al. (2019) have shown previously the value of combating deficit thinking because it has a direct link on changing teacher expectations. They show how this results in higher mathematics achievement for Māori and Pasifika learners and this is confirmed in this research.

Many teachers positively affirmed the warmth of the welcome as they went online into homes and the learning this provided them about their students. As Toni said: “They love maths and especially doing it with their family. We have been welcomed into the homes of our whānau and have learned about real-life for them.” Highlighted for the teachers was how Māori and Pasifika family and collectivist norms and values (e.g., Anae et al., 2001; Berryman & Eley, 2017; Hill et al., 2019) translated into supportive and mathematically-nourishing environments in the home. Teachers reflected on the positive aspect of having parents working alongside their children. Liz stated: “We are hearing really rich conversations and seeing parents actively supporting learning.” In this way, mathematics learning became a balanced power sharing activity as everyone engaged together and brought alive what Paris and Alim (2014) describe as asset-based pedagogy. For example, one teacher, Rita, described how one of her students was doing mathematics with her father and they both came to a different solution. Rita responded by positioning the student to have a conversation in regard to how her father had worked it out and then asking: “can you learn from your Dad how he solved it to apply it to your solution?” This example illustrates the way in which the teachers were given opportunities to walk in the world of their students and whanau and develop a different type of partnership. Other teachers highlighted how families used the time themselves to strengthen cultural connections such as language. For example, Justine shared: “one student has been playing a lot of bingo with her family each night, and her family are requiring her to say the numbers in Samoan when she’s the caller so she practises the language.” These examples all illustrated how bringing the home into school during remote learning could move some way towards what Riwai-Couch et al. (2020) proposed, in calling for a different form of home-school partnership. In this study, we share the possibilities of where changes in the boundaries between home and school within an online environment can take place.

Other learnings for the teachers occurred as they did online mathematics teaching and learning with their students. They all described the proactive nature of the parents in supporting their children’s learning. Bringing the home into the school context taught many of them that their previous assumption, that they had taken the parents on a journey of change within mathematics teaching and learning while they were implementing the DMIC professional learning and development, was erroneous. The school partnerships instead had remained traditional, which Civil and Bernier (2009) describe as needing to change. Teachers described how initially, parents often sat alongside their children and stressed rote use of basic facts and procedures. As Liz commented:

Some parents have found it hard to work on our maths problems, saying ‘maths isn’t like it used to be’. This isn’t meant in a bad way and the same parents have really enjoyed learning alongside their children. Mostly the parent sits with the student and works alongside. We can hear the parents encouraging the students to solve the problems and share thinking.

However over time, as greater balances developed in the partnership, many described the shift in parents’ perceptions of what it meant to learn and do mathematics successfully. The teachers noted that the parents adapted quickly to this different form of mathematics as they adopted different expectations of what was expected of them all in the tasks. As Kate reflected:

They [the parents] quickly adapted to realizing that the students were going to need to explain the strategy so just working it out for them or using a calculator was not going to suffice.

Mathematical talk and mathematical practices gained focus, and teachers were provided with opportunities to see possibilities for new shared understandings with parents about the nature of what it means to learn and do mathematics. Developing the form of more effective partnerships promoted by Civil and Bernier (2009) became possible.

Looking towards the future when students would return to school, all teachers and leaders were eager to find ways to continue the positive relationships they had across their home communities. As Rita said: “We have built strong working relationships between school and home which I wish to continue fostering and developing as I have seen huge growth in some students.” Clearly, they had recognized how extending the home into the school community had been empowering for them all. Crossing boundaries between homes and schools had provided possibilities for developing true power-sharing relationships as promoted by researchers (e.g., Bonner, 2014; Civil & Bernier, 2009; Hodge & Cobb, 2016; Powell et al., 2016) who argue for more equitable mathematics education. Many researchers (e.g., Adiredja, 2019; Bannister, et al., 2018; Jilk, 2016; Rogoff et al., 2017) argue that a strength-based approach has potential to address inequities in mathematics education; what this study has shown is that this does not need to happen only in schools, but can also occur in more unique settings like the lockdown presented.

## Conclusions and implications

In Aotearoa/New Zealand, many media reports voiced concern that our most impoverished group, who are predominantly Māori and Pasifika students, would be adversely affected by the loss of 2 months of schooling. In turn, we too could have taken the same deficit outlook caused by what Rubie-Davies (2016) describes as cultural mismatches. However, we knew that these students came from home environments, which although different from that of dominant cultural groups in Aotearoa/New Zealand, nonetheless had rich culturally-embedded home contexts which would afford excellent opportunities for learning mathematics. Based on the premise that the home context would differ from the school context, we set out to examine and explore the teaching and learning of mathematics when the homes of our Māori and Pasifika students become part of the school context. We also changed the focus from the mathematics learning of the students to that of their teachers and school leaders, because remote learning during the COVID-19 lockdown had reversed the roles and now teachers had to enter their students’ and their family’s spaces, rather than the reverse. Our study sought to explore two specific questions: (1) How did our participants develop and maintain culturally sustaining mathematics pedagogy during remote teaching and learning? (2) What did our participants learn as they remotely “entered” students’ homes during the pandemic? We summarize below how our findings collectively answer these two questions.

Firstly, we argue that the political focus on collective well-being in Aotearoa/New Zealand provided a pathway for schools and teachers to enact and maintain culturally sustaining pedagogy. In contrast to an individualistic focus on mathematics learning, teachers and school leadership in this study developed a culturally sustaining pedagogy that was founded on holistic relationships with students and families. This meant that rather than viewing their role as only to provide academic support, our participants also embodied actions that were guided by key Māori and Pasifika values such as service, reciprocity, and respectful relationships. This finding aligns with those of Bourke et al. (2021) who contend the importance of students being in learning spaces which are culturally congruent. Utilizing pedagogy, which prioritized well-being and built on student and family values, allowed the educators in our study to establish foundations for deeper mutual understandings and relationships with their families and students. Establishing positive relationships and trust is central to constructing culturally sustaining pedagogy (Paris & Alim, 2014), empowerment, and power sharing (Bonner, 2014; Hodge & Cobb, 2016; Powell et al., 2016) and the potential for this had become possible.

Secondly, we argue that as our participants remotely “entered” students’ homes during the pandemic, opportunities emerged for the educators to further their understanding of what culturally sustaining mathematics pedagogy entails. This novel experience allowed the educators not only to learn more about their students’ lives, but also to have their assumptions and perceptions challenged. Educators recognized the richness of relationships that were constructed as they crossed the boundaries between home and school. From this platform, educators were provided with many opportunities to see the significance of bringing the home into school. For instance, the school community were able to see the positive ways in which families supported and maintained rich home mathematics learning and the importance of recognizing and actively building on their funds of knowledge (Moll et al., 1992). Thus, a key finding of our study was that mathematics teaching and learning could and did continue within the homes of diverse learners, despite a common deficit narrative about the lack of assets at home for marginalized groups of students and how it would only be worsened by the pandemic.

Rather than the negative outcomes predicted by deficit narratives, the COVID-19 pandemic lockdown had given our teacher participants a chance to become critically conscious of the wealth that their students and whanau had to offer to the school setting. Hunter et al. (2016) and Si’ilata et al. (2018) described the benefits of teachers viewing their student’s cultural backgrounds as assets rather than deficits and evidence is provided in the present study that teachers can do this within an online learning environment. Alongside this, we also note the benefits of teachers recognizing the strengths in student and family dispositions, including the importance placed on mathematics, resilience, and students’ challenging themselves mathematically. In this sense, the hopes of Riwai-Couch et al. (2020) for true partnerships between home and school hold promise.

An enduring theme emphasized by all teachers and leaders in our study was the hope that they could continue the close power-sharing relationships and learnings the COVID-19 lockdown had afforded them of their students and their whānau in the busy everyday life of teaching mathematics in school. What would this take? This needs to be considered in the new post COVID-19 world, in which new ways of thinking and doing are possible. We have highlighted in this article that although the COVID-19 pandemic has caused disruption and distress, we can also use it as an opportunity to grow and reimagine mathematics education for the future.

## Data Availability

The data that support the findings of this study are available on reasonable request from the corresponding author (Jodie Hunter). The data are not publicly available due to them containing information that could compromise research participant privacy.
